# 

**Published:** 1904-05

**Authors:** 


### BOOKS RECEIVED.

Manual of Clinical Microscopy and Chemistry for Students and Practitioners of Medicine. By Dr. Hermann Lenhartz, Professor of Medicine and Director of Hospital at Hamburg, etc. Authorised Translation from the Fourth and Last German Edition, with Notes and Additions, by Henry T. Brooks, M. D., Professor of Histology and Pathology at the New York Post-Graduate Medical School and Hospital. With 148 illustrations and 9 colored plates. Pages xxxii-412. Octavo. F. A. Davis Company. Philadelphia, Pa. 1904. (Price, $3.00 net.)

A Textbook of Physiology. By Isaac Ott, A. M., M. D., Professor of Physiology in the Medico-Chirurgical College of Philadelphia. With 137 illustrations. Octavo, 563 pages. F. A. Davis Company, Philadelphia, Pa. 1904. (Price, $3.00 net.)

Transactions of the College of Physicians of Philadelphia. Third series. Volume twenty-fifth. Edited by William Zentmayer, M. D. Philadelphia: W. J. Dornan, Printer. 1903.

Annual Report of the Surgeon-General of the Public Health and Marine Hospital Service of the United States. For the fiscal year 1902. Washington: Government Printing Office. 1903.

Medical Union Number Six. By William Harvey King. Sextodecimo, 60 pages. The Monograph Press, New York. 1904. (Price, boards, 35.)

